# Multi-Dose Priming Regimens of PfSPZ Vaccine: Safety and Efficacy against Controlled Human Malaria Infection in Equatoguinean Adults

**DOI:** 10.4269/ajtmh.21-0942

**Published:** 2022-02-07

**Authors:** Said Abdallah Jongo, L. W. Preston Church, Vicente Urbano Nsue Ndong Nchama, Ali Hamad, Raul Chuquiyauri, Kamaka Ramadhani Kassim, Thabit Athuman, Anna Deal, KC Natasha, Ali Mtoro, Maxmillian Mpina, Elizabeth Nyakarungu, Gertrudis Owono Bidjimi, Marta Alene Owono, Escolástica Raquel Mansogo Mayé, Martín Eká Ondó Mangue, Genaro Nsué Nguema Okomo, Beltrán Ekuá Ntutumu Pasialo, Dolores Mbang Ondó Mandumbi, María-Silvia A. López Mikue, Fortunata Lobede Mochomuemue, Mariano Obiang Obono, Juan Carlos Momo Besahá, José Raso Bijeri, Gabriel Mbá Abegue, Yolanda Rimoy Veri, Ines Toichoa Bela, Federico Comsil Chochi, José Enrique Lima Sánchez, Vanessa Pencelli, Griselda Gayozo, José Antonio Esono Mbá Nlang, Tobias Schindler, Eric R. James, Yonas Abebe, Laurence Lemiale, Thomas C. Stabler, Tooba Murshedkar, Mei-Chun Chen, Christopher Schwabe, Josea Ratsirarson, Matilde Riloha Rivas, Mitoha Ondo’o Ayekaba, Diosdado Vicente Nsué Milang, Carlos Cortés Falla, Wonder P. Phiri, Guillermo A. García, Carl D. Maas, Bonifacio Manguire Nlavo, Marcel Tanner, Peter F. Billingsley, B. Kim Lee Sim, Claudia Daubenberger, Stephen L. Hoffman, Salim Abdulla, Thomas L. Richie

**Affiliations:** ^1^ Bagamoyo Research and Training Center, Ifakara Health Institute, Bagamoyo, Tanzania;; ^2^ Sanaria Inc., Rockville, Maryland;; ^3^ Ministry of Health and Social Welfare, Equatorial Guinea, Malabo, Equatorial Guinea;; ^4^ Clinical Immunology Unit, Department of Medical Parasitology and Infection Biology, Swiss Tropical and Public Health Institute, Basel, Switzerland;; ^5^ University of Basel, Basel, Switzerland;; ^6^ Protein Potential LLC, Rockville, Maryland;; ^7^ Medical Care Development International (MCDI), Silver Spring, Maryland;; ^8^ Marathon EG Production, Ltd., Bioko Norte, Equatorial Guinea

## Abstract

*Plasmodium falciparum* sporozoite (PfSPZ) Vaccine is composed of radiation-attenuated, aseptic, purified cryopreserved PfSPZ. Multiple clinical trials empirically assessing two to six doses have shown multi-dose priming (two to four doses the first week) to be optimal for protection in both 4- and 16-week regimens. In this randomized, double-blind, normal saline (NS) placebo-controlled trial, four groups (G) of 18- to 32-year-old Equatoguineans received multi-dose priming regimens with or without a delayed final dose at 4 or 16 weeks. The regimens were G1: days 1, 3, 5, 7, and 113; G2: days 1, 3, 5, and 7; G3: days 1, 3, 5, 7, and 29; and G4: days 1, 8, and 29. All doses were 9 × 10^5^ PfSPZ. Tolerability, safety, immunogenicity, and vaccine efficacy (VE) against homologous controlled human malaria infection (CHMI) 6–7 weeks after vaccination were assessed to down-select the best regimen. All four regimens were safe and well tolerated, with no significant differences in adverse events (AEs) between vaccinees (*N* = 84) and NS controls (*N* = 20) or between regimens. Out of 19 controls, 13 developed Pf parasitemia by quantitative polymerase chain reaction (qPCR) after CHMI. Only the vaccine regimen administered on study days 1, 8, and 29 gave significant protection (7/21 vaccinees versus 13/19 controls infected, VE 51.3%, *P* = 0.03, Barnard’s test, two-tailed). There were no significant differences in antibodies against Pf circumsporozoite protein (PfCSP), a major SPZ antigen, between protected and nonprotected vaccinees or controls pre-CHMI. The six controls not developing Pf parasitemia had significantly higher antibodies to blood stage antigens Pf exported protein 1 (PfEXP1) and Pf merozoite surface protein 1 (PfMSP1) than the controls who developed parasitemia, suggesting naturally acquired immunity against Pf limited infections in controls. This study identified a safe, protective, 4-week, multi-dose prime vaccination regimen for assessment in future trials of PfSPZ Vaccine.

## INTRODUCTION

Malaria continues to be a major global health problem, with the WHO African Region reporting 215 million cases (94% of the global burden) in 2019. Malaria incidence rates worldwide have remained static at ∼57 cases per 1,000 population at risk since 2015. ^1^ The increasing prevalence of molecular markers of artemisinin resistance and detection of resistance to all main insecticide classes throughout sub-Saharan Africa threaten control measures currently deployed. If the goal of malaria elimination is to be achieved, additional tools are needed, including vaccines that can prevent infection and thereby block transmission.

Sanaria^®^ PfSPZ Vaccine (Sanaria Inc., Rockville, MD), composed of radiation-attenuated, aseptic, purified, cryopreserved, whole *Plasmodium falciparum* (Pf) sporozoites (SPZ), is designed to achieve these objectives. PfSPZ Vaccine has been assessed in 21 completed or ongoing trials in the United States, European Union, and Africa, and shown to be safe and well tolerated, ^2^^–^ ^17^ with almost no differences in adverse event (AE) profiles between vaccinees and normal saline (NS) placebo recipients in 12 of the 13 trials among the 21 that used a randomized, double-blind, placebo-controlled design (in one trial conducted in Burkina Faso, there was an increased frequency myalgia in vaccinees—Sirima and Laurens, unpublished). Vaccine efficacy (VE) > 90% against homologous (same parasite strain in vaccine and challenge) controlled human malaria infection (CHMI) at 3–11 weeks after last dose has been shown in the United States, ^3^^,^ ^5^ Tanzania, ^11^ and Mali, ^17^ and can last for at least 14 months. ^4^ In field studies, during 24 weeks of follow-up post-vaccination in three trials in Mali and one in Burkina Faso, VE against first episode of parasitemia ranged from 48% to 57% by time-to-event analysis (one minus the hazard ratio) ^7^^,^ ^17^ (Sirima and Laurens, unpublished; Diawara and Healy, unpublished), and in both Burkina Faso and the most recent Mali trial, VE against infection was sustained during a second malaria transmission season, as was VE against clinical malaria in Mali (Sirima and Laurens, unpublished; Diawara and Healy, unpublished). Vaccine efficacy against clinical malaria has also been demonstrated in Kenyan infants. ^16^

The most promising VE against CHMI has been seen with vaccine doses of 9.0 × 10^5^, with protection reduced when higher doses were tested in malaria-exposed Tanzanian ^11^ and Equatoguinean ^14^ adults. However, optimal timing and number of doses have not been defined. Previous studies tested longer vaccination schedules (three to five doses administered over 16–20 weeks), with relatively even spacing. ^2^^–^ ^11^^,^ ^13^^–^ ^15^^,^ ^17^ Recent data indicate that accelerated vaccination schedules, particularly for the initial priming immunizations (multi-dose priming), allow shortening of the vaccination period and may provide better VE. For example, in a study in the United States (Warfighter 2 trial), a five-dose regimen consisting of four priming doses over the first 6 days (days 1, 3, 5, and 7) with a boost at 16 weeks using 4.5 × 10^5^ PfSPZ per dose gave significant VE (40%) against heterologous CHMI (different Pf strains in the vaccine and challenge), whereas three regimens of three evenly spaced administrations over 16 weeks using higher doses (0.9, 1.8, and 2.7 × 10^6^ PfSPZ) did not provide significant VE (20–23%). ^15^ A subsequent study in Germany (MAVACHE trial) showed that the VE was 77% and 79% against homologous and heterologous CHMI, respectively, when the boost was administered on day 29; however, both a multi-dose prime (two doses on days 1 and 8) and the boost were required, as just two administrations of the same (9.0 × 10^5^) or higher (1.35 or 2.7 × 10^6^) doses of PfSPZ protected only 67% and 50%, respectively (Mordmüller, unpublished). A condensed administration schedule of PfSPZ Vaccine maintaining high VE such as that tested in Germany would facilitate its deployment in the field.

Here, we report the results of a randomized, double-blind, NS placebo-controlled clinical trial (EGSPZV3) assessing the safety, immunogenicity, and VE of four different multi-dose priming regimens with or without a delayed booster dose, each dose containing 9.0 × 10^5^ PfSPZ of PfSPZ Vaccine administered by direct venous inoculation (DVI). Two of the regimens tested, both described above, had been the best among several evaluated in two prior regimen-optimization trials. To better compare these two leading regimens, two bridging groups were added in this study to assess the effect of the interval prior to booster dose (15 weeks in the first leading regimen, 3 weeks in the second) and to examine the need for a booster dose.

The trial enrolled healthy, malaria-exposed adults living on Bioko Island in Equatorial Guinea. We selected this population rather than malaria-naive individuals because the most important application planned for PfSPZ-based vaccines is their use in mass vaccination programs (MVPs) in endemic areas to regionally eliminate malaria transmission. It was therefore appropriate to conduct this regimen-comparison study in the target population, although the ethical concerns associated with conducting CHMI in pediatric age-groups limited participation to adults. ^18^ Vaccine efficacy was assessed 6–7 weeks following final immunization by homologous CHMI. The trial was designed to identify an optimal immunization regimen for further testing in Phase 2 and 3 clinical trials.

## MATERIALS AND METHODS

### Study location and population.

This single-center trial was conducted at the Baney Clinical Research Center located near the capital city Malabo on Bioko Island between August 2018 and March 2019. Healthy male and female adults of age 18–45 years were recruited from the Baney and Rebola districts and Malabo. After successfully completing a test of study understanding and signing informed consent forms (ICFs), prospective participants were screened and enrolled according to inclusion and exclusion criteria (Supplemental Tables 4 and 5; https://clinicaltrials.gov/show/NCT03590340). Individuals testing positive for HIV, hepatitis C, or hepatitis B were excluded from participation. Women of childbearing potential were required to use injectable depot hormonal contraception.

### Investigational products.

The investigational product (IP), Sanaria^®^ PfSPZ Vaccine, consists of radiation-attenuated (metabolically active but nonreplicating), aseptic, purified, vialed, cryopreserved PfSPZ and is stored in liquid nitrogen vapor phase at −150 to −196°C. ^19^ After thaw, vialed PfSPZ were diluted in phosphate-buffered saline and human serum albumin in a biological safety cabinet, and injected within 30 minutes. Normal saline was used as a neutral comparator for assessment of safety as it is visibly indistinguishable from the vaccine, can be administered by DVI, and is not associated with AEs. PfSPZ Vaccine or NS in 0.5 mL was administered by DVI through a 25-gauge needle over several seconds. Sanaria^®^ PfSPZ Challenge (NF54), used for CHMI, was manufactured, stored, and administered identically to PfSPZ Vaccine apart from the attenuation (irradiation) step. When administered by DVI at the standard dose of 3.2 × 10^3^ PfSPZ, PfSPZ Challenge has led to patent parasitemia in 100% of 78 malaria-naive adults ^20^^–^ ^29^ and 6/7 Equatoguineans ^14^ on first CHMI.

### Randomization and intervention.

**Figure 1.**
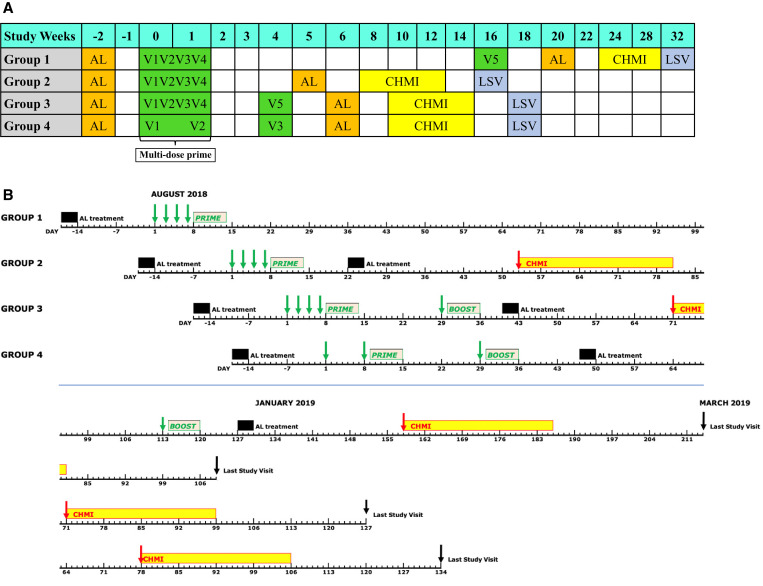
Schema and execution plan for EGSPZV3 clinical trial. (**A**) Shows the design of the four study groups. Group 1 was the best regimen from the Warfighter 2 clinical trial, ^15^ Group 4 was the best regimen from the MAVACHE trial (Mordmüller, unpublished), and Groups 2 and 3 were bridging groups to explore the importance of the boost and the length of the prime-boost interval. AL = artemether/lumefantrine; V1–V5 = vaccinations 1–5; CHMI = controlled human malaria infection; LSV = last study visit. Minimum days between AL and V1: 14 days. Minimum days between AL and CHMI: 23 days. (**B**) Shows the execution plan for the study. Green arrows: immunizations; red arrows: CHMI; black arrows: last study visits. This figure appears in color at www.ajtmh.org.

The 104 participants were allocated into four groups of 26 participants (Figure 1). In all groups, participants were randomized to receive PfSPZ Vaccine (*N* = 21 per group, 9.0 × 10^5^ PfSPZ per dose) or NS (*N* = 5 per group). In Groups 1–3, 9.0 × 10^5^ PfSPZ were administered four times on days 1, 3, 5, and 7 followed by 1) a final dose on day 113/week 16 (regimen 1 as previously studied in the Warfighter 2 trial, NCT02601716 ^15^), 2) no final dose (regimen 2, a bridging group to examine the need for a final dose), or 3) a final dose on day 29/week 4 (regimen 3, a bridging group with a shorter interval to the final dose to match regimen 4). In regimen 4, 9.0 × 10^5^ PfSPZ were administered on days 1 and 8 with a final dose on day 29 (best regimen from MAVACHE trial, NCT02704533). Although group assignment was unblinded, participants, clinical staff, and study outcome assessors were blinded to treatment assignment within each group. Because parasitemia is immunosuppressive ^30^^–^ ^32^ and appears to inhibit the ability of PfSPZ to induce a protective immune response, ^28^^,^ ^33^^,^ ^34^ all participants received a full treatment course of AL (artemether 80 mg/lumefantrine 480 mg) twice a day for 3 days under directly observed treatment (DOT) prior to first immunization, allowing at least 14 full days between the last drug dose and the first immunization. The same regimen was used to treat any malaria infections acquired through natural transmission during the study.

### Vaccine efficacy.

Vaccine efficacy was assessed by homologous CHMI of 3.2 × 10^3^ PfSPZ of PfSPZ Challenge (NF54) administered by DVI. Controlled human malaria infection was targeted for 8 weeks after the last immunization for all participants. To prevent the confounding of VE assessment by naturally acquired Pf infection, given that the study was located in an area of active malaria transmission, participants completed a second full treatment course of AL under DOT approximately 3 weeks after final immunization and at least 23 days prior to CHMI to minimize the possibility of residual drug effects at the time of CHMI. Twenty-three days is four to eight times the 3- to 6-day half-life of the longer acting drug partner, lumefantrine. After PfSPZ Challenge injection, participants were observed for 30 minutes and then discharged from the clinic. Follow-up for parasitemia and for signs and symptoms of malaria began on day 6 after CHMI, performed on an outpatient basis (days 6 and 7) and then inpatient basis (day 8 onward) using thick blood smear (TBS), quantitative polymerase chain reaction (qPCR), and clinical assessment until day 21 or until Pf parasitemia was diagnosed and treatment of malaria with AL completed, whichever came first. Samples for TBS and qPCR were obtained daily on days 6 and 7, every 12 hours beginning on day 8 through day 14, then daily from day 15 through day 21. Any positive TBS was confirmed by qPCR by an independent technician prior to treatment. All other qPCR testing was performed retrospectively. Participants remaining TBS negative on day 21 continued with alternate-day outpatient monitoring until day 28. Thick blood smear sampling could be performed more frequently at the discretion of study investigators if volunteers had symptoms or signs consistent with malaria. After initiation of treatment, TBS was assessed daily until two consecutive samples were negative by TBS. Individuals who dropped out of the study prior to day 28 without a malaria diagnosis and those who remained negative on day 28 were pre-emptively treated with AL and confirmed negative by qPCR. A sample for qPCR was obtained at the final scheduled study visit (42 days after CHMI) to confirm that no study participants were left with a residual malaria infection at the end of the study.

### Adverse events assessment.

Solicited AEs following inoculation were assessed utilizing a prespecified list of signs and symptoms (Supplemental Table 6) for 2 days (local AEs) or 7 days (systemic AEs) after each injection using in-person clinic or telephone visits and were rated for severity. All solicited AEs were considered related to IP administration and attributed to the preceding injection. Solicited AEs following administration of PfSPZ Challenge for CHMI were recorded utilizing the same prespecified criteria as for PfSPZ Vaccine or NS injection, except that the list of AEs was expanded on day 6 post CHMI to include additional symptoms that might indicate clinical malaria and collection of systemic AEs continued until the diagnosis and treatment of Pf parasitemia, or until day 28, whichever came first. Solicited AEs recorded through 5 days after CHMI were attributed to PfSPZ Challenge, and from day 6 onward to parasitemia (Supplemental Table 6).

Unsolicited AEs were assessed by open-ended questioning and recorded from day 1 to day 14 after each injection or series of multi-dose prime injections and from day 1 to day 28 after CHMI and were rated for severity. During the first 5 days after CHMI start, unsolicited AEs were considered potentially related to PfSPZ Challenge, and thereafter through to day 28 to asexual blood stage parasitemia. A relatedness attribution to parasitemia was kept only if the subject turned out to be positive for Pf by TBS or qPCR.

Hematology and biochemistry testing was performed 14 days after the priming regimen and the final dose; abnormal results were rated for severity and clinical significance (Supplemental Table 7).

### Detection of Pf parasites and parasite DNA.

Subjects had scheduled assessments for parasitemia by TBS at four time points outside of CHMI: at screening, and prior to the first dose, the final dose and CHMI (for Group 2, which did not receive a delayed final dose, the third sample was taken 2 weeks after the final dose of the priming regimen). Except at screening, all positive TBS were confirmed retrospectively by qPCR after the conclusion of the study. Additional TBS were to be made for any symptomatic individual.

Slide preparation and reading for TBSs were performed as described. ^35^ In brief, 10 μL of blood collected in ethylenediaminetetraacetic acid were evenly spread over a 10 mm × 20 mm rectangle on a glass slide, dried, and stained with Giemsa. For asymptomatic individuals, ∼0.5 μL of blood were assessed. For symptomatic individuals, ∼2.0 μL of blood were assessed. Two asexual erythrocytic stage Pf parasites had to be identified for a sample to be considered positive; thus, the theoretical lower limit of detection for a positive TBS was 4 parasites/μL blood in an asymptomatic subject and 1 parasite/μL in a symptomatic subject. Subjects were monitored by qPCR until day 28 after CHMI or until malaria treatment based on TBS positivity.

Parasites were quantified by qPCR using the PlasQ qPCR assay that has a lower limit of dection of 50 parasites/mL, as described. ^36^ A single positive result was considered positive for infection with Pf. After the start of CHMI, the time of first blood sample positivity by qPCR was used to determine infection status and calculation of prepatent period.

### Antibody assays.

Blood for antibody testing was drawn at baseline prior to the first immunization, 2 weeks after the final immunization and prior to CHMI. Serum was separated and frozen at −80°C within 4 hours of collection. IgG antibodies to Pf circumsporozoite protein (PfCSP), Pf merozoite surface protein 1 (PfMSP1), and Pf exported protein 1 (PfEXP1) were assessed by ELISA as described. ^10^ Samples for PfCSP were considered positive (seroconversion) if the difference between the postimmunization optical density (OD) 1.0 and the preimmunization OD 1.0 (net OD 1.0) was > 50 and the ratio of postimmunization OD 1.0 to preimmunization OD 1.0 (ratio) was > 3.

### Statistics.

The sample size of 21 subjects in each vaccine group with a pooled total of 20 subjects receiving NS was selected to have a 90% power to detect a VE of 35–50% for each vaccine group compared with the controls, allowing for a 20% dropout rate from each group and assuming that no more than one control subject would fail to develop detectable parasitemia. Categorical variables were summarized using absolute (*n*) and relative (%) frequencies. Continuous variables were summarized using mean and SD, median, and range. Comparisons of categorical variables between groups were analyzed using Barnard’s two-sided exact unconditional test; for comparisons of continuous variables including differences between vaccinees and controls in the results of antibody assays were analyzed using the Mann–Whitney two-sided test. Vaccine efficacy was estimated as 1—(attack rate in vaccine subjects/attack rate in NS control subjects) based upon parasitemia detected by qPCR. The immune responses of study subjects in each group were compared with the immune responses of the pooled placebo group. Within each group, immune responses in subjects protected were compared with the immune responses in subjects not protected against CHMI. For antibody assays, differences between vaccinees and controls were analyzed using the Mann–Whitney test for net OD 1.0 and OD 1.0 ratios. No adjustments were made for multiple comparisons.

### Study approval.

Ethical approval was obtained from the National Ethics Committee of Equatorial Guinea and the Ifakara Health Institute Institutional Review Board. The volunteers were briefed on the specifics of the planned study, had to pass a written assessment of understanding of the study, and signed informed consent before any study procedures were done.

## RESULTS

**Figure 2.**
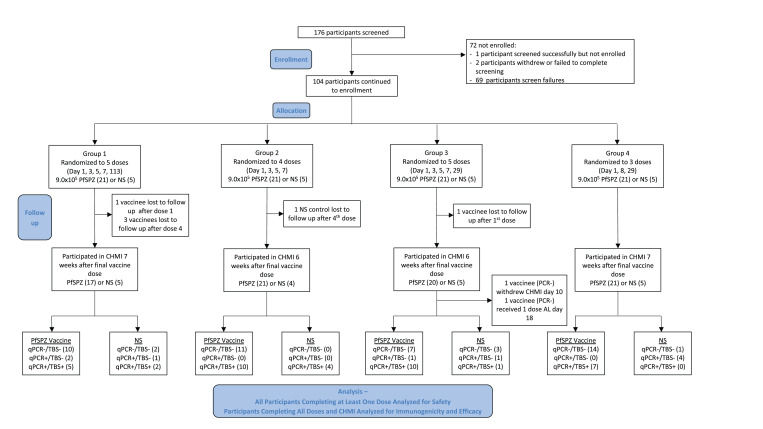
CONSORT flow diagram. This figure appears in color at www.ajtmh.org.

A total of 104 subjects (91 male, 13 female) aged 18–32 years were enrolled and allocated to one of the four study groups. Twenty-six subjects in each group were randomized to receive vaccine (*N* = 21) or NS (*N* = 5) (Figure 2). Vaccinees in each group were well balanced with respect to age and body mass index (BMI) when compared with each of the other groups or the pooled control groups (Supplemental Table 1). Few women were enrolled in the study—a consequence of the requirement for stringent pregnancy prevention that many women refused. All 13 female subjects were randomized by chance to receive the vaccine. Five subjects withdrew or were lost to follow-up prior to CHMI—four from Group 1, which had the longest dosing period (113 days) and one from Group 3; all five had been randomized to receive PfSPZ Vaccine.

### Parasitemia in subjects prior to CHMI.

No volunteer was symptomatic for malaria during the preimmunization and immunization phases of the study and thus no blood smears were performed other than at protocol-specified time points of screening, prior to first vaccination, prior to final dose (or 2 weeks after the fourth priming dose in the case of Group 2), and prior to CHMI. TBS and qPCR results from these timepoints are presented in Supplemental Table 2.

Five participants were positive by TBS at screening among those later enrolled in the trial—one in Group 1 and two each in Groups 3 and 4. All subjects were treated presumptively with AL after screening and prior to V1 as stipulated by the protocol. At the next time point, prior to V1, one subject (Group 3, NS control) was TBS positive (confirmed by qPCR, density of 1.84 genome copies/μL) 14 days after the last dose of AL and was treated again with AL, with immunization deferred 3 weeks. A second participant (Group 1, PfSPZ Vaccine) was retrospectively qPCR positive 16 days after the last dose of AL (1.31 genome copies/μL) and may have been parasitemic at V1. Both subjects likely acquired malaria by natural transmission.

At the time of the last vaccine dose, 38–126 days after being treated with AL, one participant was positive by TBS and this subject plus four more were found positive by qPCR, indicating that they had probably acquired Pf infections by natural transmission during the immunization phase. Among these five subjects, two were vaccinees from Group 4 (densities of 0.39 genome copies/μL blood and 0.70 genome copies/μL blood), two were controls from Group 3 (16.31 genome copies/μL blood and 227.4 genome copies/μL blood), and one, with a density of 6,740 genome copies/μL blood, was a NS control from Group 4. This last subject, although asymptomatic, had a positive TBS, was treated with AL and received the final injection 41 days after treatment, this constituting a 20-day delay in the final dose. The other four may therefore have been parasitemic at the time they received their final dose. All subjects were subsequently presumptively treated with AL 24–29 days before CHMI as stipulated by the protocol and no subjects were TBS or PCR positive prior to CHMI.

All three vaccinees and three of the four controls identified as qPCR positive during the immunization period remained negative during CHMI follow-up. Only the control from Group 4 whose TBS had been positive prior to V3 developed parasitemia after CHMI (day 20).

### Vaccine efficacy.

#### Normal saline controls.

Out of 20 controls, 19 participated in one of the four CHMIs conducted 6–7 weeks after the final dose of NS, which was within the protocol-defined window. One control subject was lost to follow-up after last injection and did not participate. Out of 19 controls, 13 were positive by qPCR after CHMI (seven of these positive by TBS) and six controls were negative by qPCR and TBS. Results for the controls were pooled for comparison to each of the vaccine groups (Table 1). All subjects who tested negative throughout the 28 days of observation were presumptively treated on day 28 with AL and confirmed qPCR negative on the last visit day 42.

**Table 1:** VE against homologous CHMI

		# Completing CHMI per protocol	Median time from last vaccine dose to CHMI (range)	# Without parasitemia at 28 days by		VE by qPCR*
				TBS	qPCR	
PfSPZ Vaccine	Group 1 (1, 3, 5, 7, 113)	17	52 days	12	10 (58.8%)	39.8% (*P* = 0.13)
	Group 2 (1, 3, 5, 7)	21	46 days (42–46 days)	11	11 (52.4%)	30.4% (*P* = 0.22)
	Group 3 (1, 3, 5, 7, 29)	18†‡	42 days	8	7 (38.9%)	10.7% (*P* = 0.74)
	Group 4 (1, 8, 29)	21	48 days	14	14 (66.7%)	51.3% (*P* = 0.03)
Controls	Pooled	19	–	12	6 (31.6%)	–
Total		96		57	48	

CHMI = controlled human malaria infection; qPCR = quantitative polymerase chain reaction; TBS = thick blood smear; VE = vaccine efficacy.

* VE calculated as VE = one-risk ratio. *P* values calculated using Barnard’s test, two-tailed.

† One subject withdrew from inpatient observation on day CHMI+10 for personal reasons, was treated and was not included in VE calculations or counted in the 18 completing CHMI.

‡ One subject was unintentionally treated with single dose of AL on day CHMI+18. This subject remained negative throughout the duration of CHMI follow-up but was not included in VE calculations or counted in the 18 completing CHMI.

#### PfSPZ Vaccine, Group 1.

**Figure 3.**
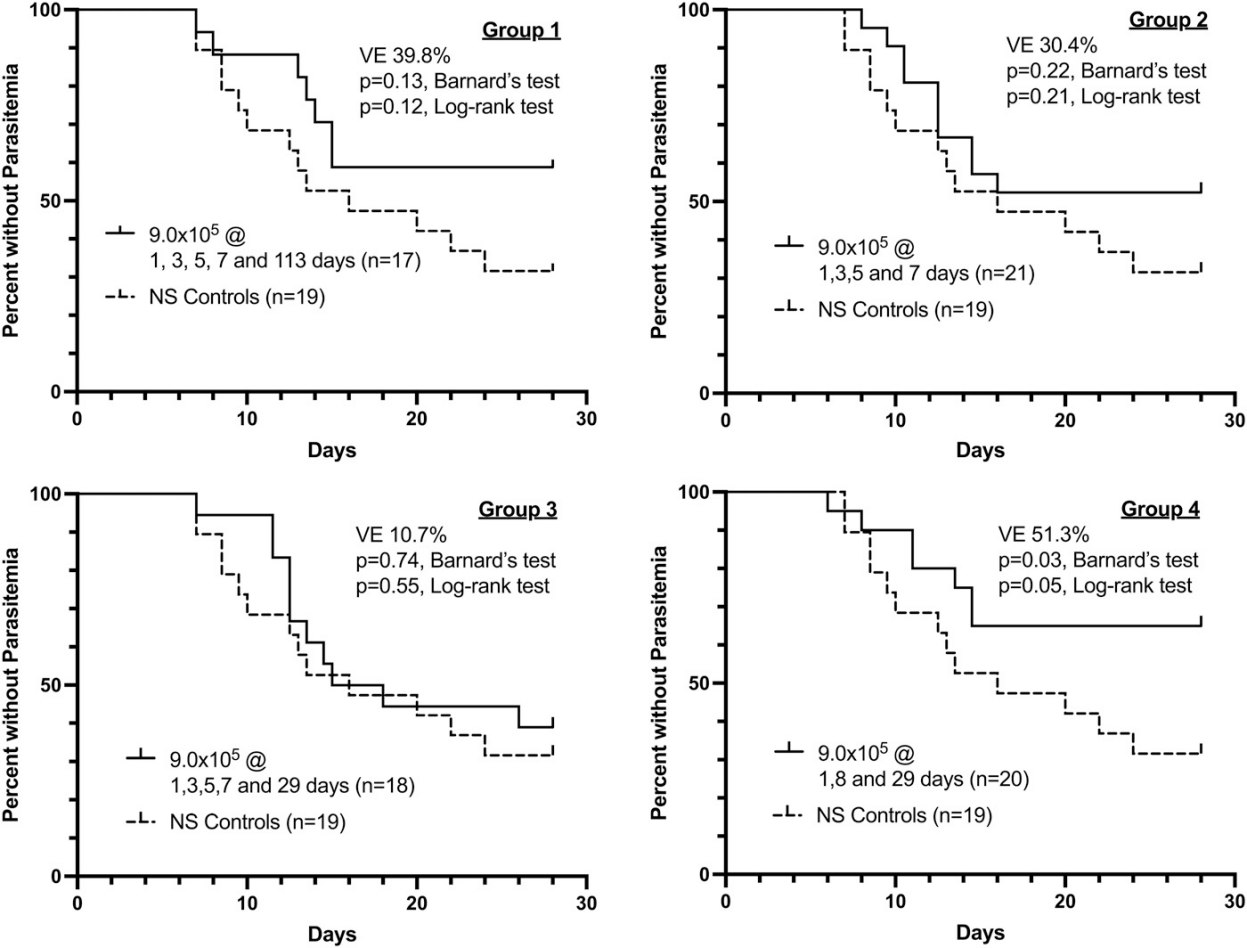
Kaplan–Meier survival curves in vaccinees and controls as assessed by quantitative polymerase chain reaction (qPCR).

Out of 21 subjects, 17 were immunized with five doses of 9.0 × 10^5^ PfSPZ of PfSPZ Vaccine on days 1, 3, 5, 7, and 113, and underwent CHMI 7 weeks after the last vaccine dose. One subject was lost to follow-up after the first immunization and three subjects traveled outside the study area after the fourth immunization. Seven of 17 subjects were positive by qPCR (5/7 positive by TBS) and 10/17 were negative by both qPCR and TBS (Table 1). Vaccine efficacy at 7 weeks after last dose of vaccine was 39.8% (*P* = 0.13, Barnard’s test, two-tailed) (Figure 3).

#### PfSPZ Vaccine, Group 2.

A total of 21 subjects were immunized with four doses of 9.0 × 10^5^ PfSPZ of PfSPZ Vaccine on days 1, 3, 5, and 7, and underwent CHMI 6 weeks after last vaccine dose. Out of 21 subjects, 10 were positive by qPCR (10/10 positive by TBS) and 11/21 were negative by both qPCR and TBS (Table 1). Vaccine efficacy at 6 weeks after last dose of vaccine was 30.4% (*P* = 0.22) (Figure 3).

#### PfSPZ Vaccine, Group 3.

A total of 20 subjects were immunized with five doses of 9.0 × 10^5^ PfSPZ of PfSPZ Vaccine on days 1, 3, 5, 7, and 29, and underwent CHMI 6 weeks after last vaccine dose. Two subjects, both recipients of PfSPZ Vaccine, were excluded from per protocol analysis. One subject, persistently TBS and qPCR negative to day 18 after CHMI, was unintentionally given a single dose of AL on day 18; this subject remained negative for the duration of the study. The other subject declined continued participation in the inpatient phase of the study on day 10 after CHMI. This subject, who was qPCR and TBS negative on day 10, was treated preemptively with AL and discharged with safety visits on post CHMI days 28 and 42. Out of 18 remaining subjects, 11 were positive by qPCR (10/11 positive by TBS) and seven were negative by both qPCR and TBS (Table 1). Vaccine efficacy at 6 weeks after last dose of vaccine was 10.7% (*P* = 0.74) (Figure 3).

#### PfSPZ Vaccine, Group 4.

A total of 21 subjects were immunized with three doses of 9.0 × 10^5^ PfSPZ of PfSPZ Vaccine on days 1, 8, and 29, and underwent CHMI 7 weeks after last vaccine dose. Seven were positive by qPCR (7/7 positive by TBS) and 14 were negative by both qPCR and TBS (Table 1). Vaccine efficacy at 7 weeks after last dose of vaccine was 51.3% (*P* = 0.03) (Figure 3).

#### Male versus female.

There were no differences in VE among the men and women undergoing CHMI (data not shown).

### Antibody responses.

#### Antibodies to PfCSP.

**Figure 4.**
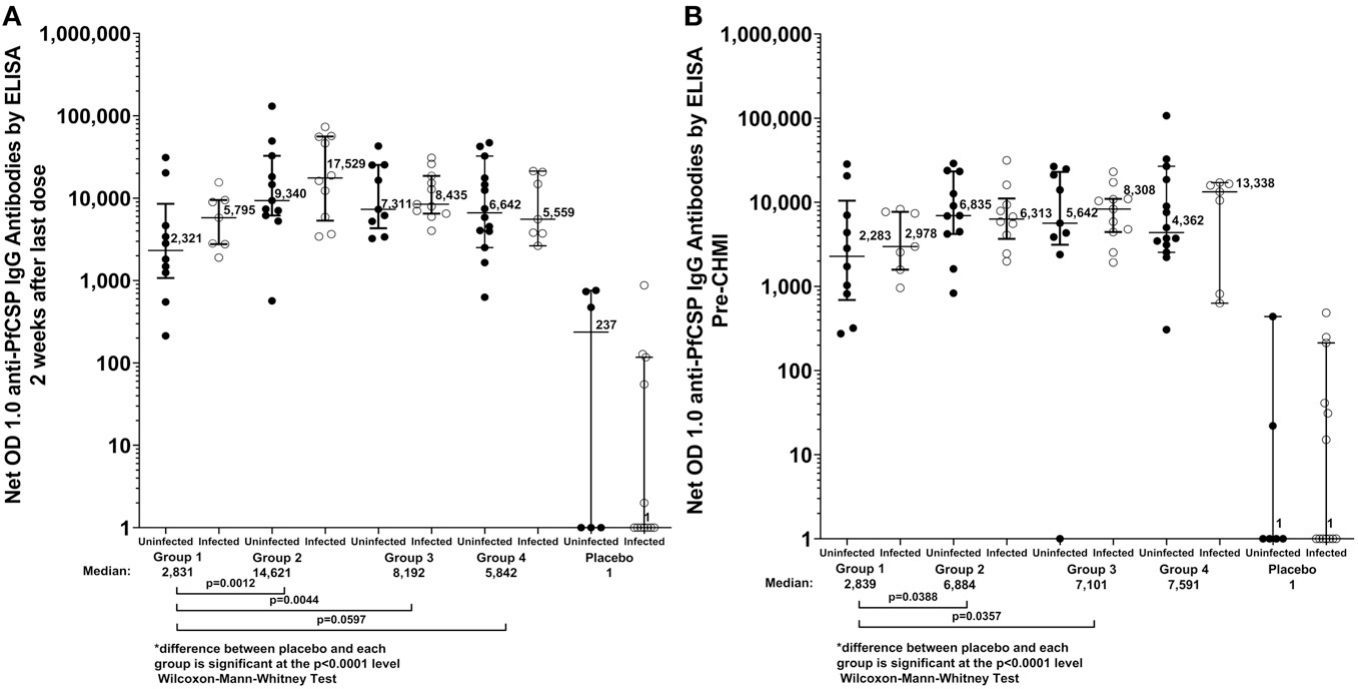
Antibodies to *Plasmodium falciparum* circumporozoite protein (PfCSP) by ELISA 2 weeks after the final vaccine dose (**A**) and immediately prior to controlled human malaria infection (CHMI) (6–7 weeks after the final vaccine dose) (**B**). For each group results are paired by subjects who were not infected (•) or infected (^) after CHMI.

IgG antibodies against PfCSP measured by ELISA were assessed in participants from all groups prior to the first immunization, 2 weeks after the final immunization, and prior to CHMI. For each vaccine group, the median net OD 1.0 at 2 weeks after the final dose and 6 weeks after the final dose (prior to CHMI) were significantly higher than the median net OD 1.0 for the control subjects (Figure 4, *P* < 0.0001 for all comparisons, Wilcoxon–Mann–Whitney test). In the three groups who received vaccine doses at days 1, 3, 5, and 7, the median net OD 14 days later was similar for all three groups (Supplemental Figure 1) and was slightly lower 14 days after the fifth (final) dose in Groups 1 and 3. For the subjects within each vaccine group who received PfSPZ Vaccine, and separately for the pooled control group, there was no significant difference in median net OD 1.0 between the infected and uninfected subjects at either the post-immunization or pre-CHMI timepoint (*P* > 0.25 for all comparisons).

#### Antibodies to PfEXP1 and PfMSP1.

**Figure 5.**
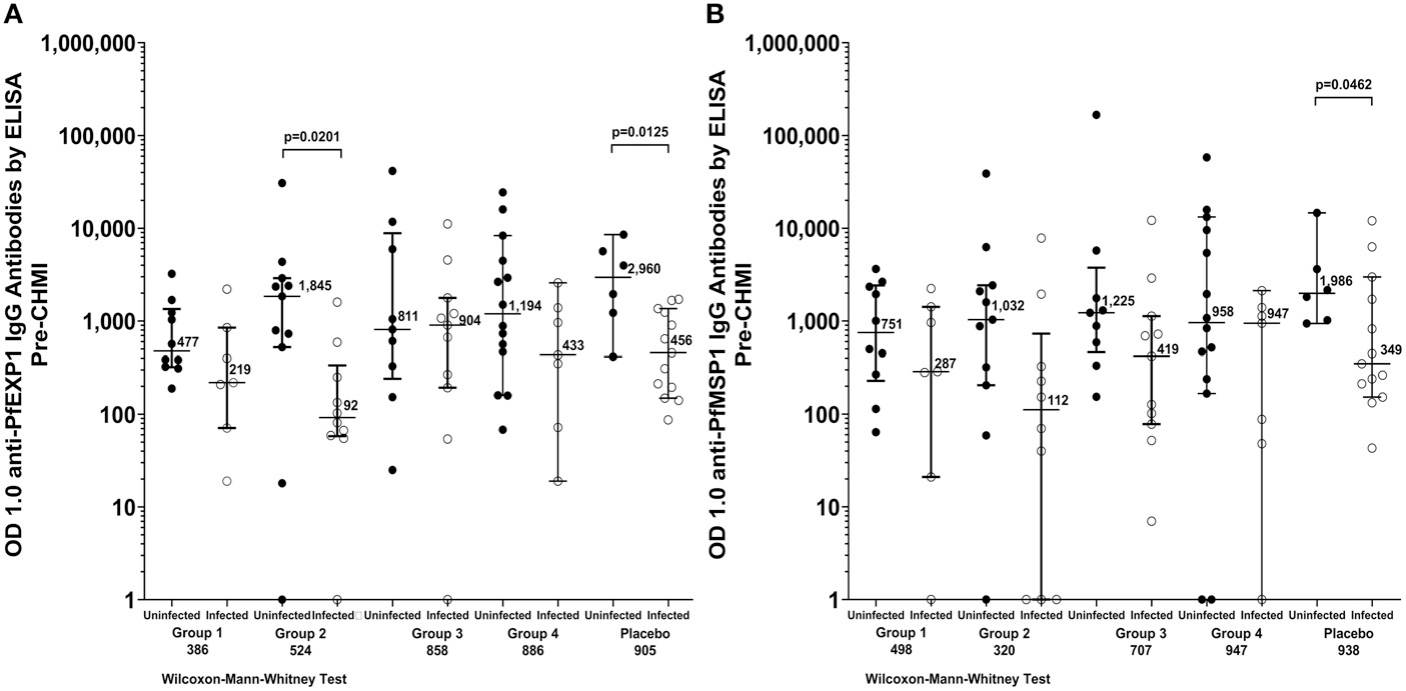
Antibodies to Pf exported protein 1 (PfEXP1) (**A**) and Pf merozoite surface protein 1 (PfMSP1) (**B**) by ELISA prior to controlled human malaria infection (CHMI). For each group, results are paired by subjects who were not infected (•) or infected (○) after CHMI.

IgG antibodies against PfMSP1 and PfEXP1 were assessed in participants from all groups prior to CHMI (Figure 5). In vaccine Groups 1, 2, and 4, and in the control group, the median antibody level to PfEXP1 was higher in subjects who were uninfected after CHMI; this difference was statistically significant for Group 2 and for the NS control group. The median antibody level to PfMSP1 was also higher in uninfected subjects for each group; the difference was statistically significant only for the NS control group. All antibody responses from the trial are provided in Supplemental Table 3.

### Safety.

#### Solicited AEs following immunization.

No SAEs or grade 3 AEs, solicited or unsolicited, were reported by any subject participating in this trial prior to CHMI. Out of 84 vaccinees, 12 (14%) experienced 14 grade 1 local AEs compared with two (10%) NS controls who experienced three grade 1 local AEs (*P* = 0.69, Barnard’s test, two-tailed) (Table 2). Nine of 84 (11%) vaccinees experienced 14 systemic AEs (12 grade 1, 2 grade 2) compared with no NS controls (*P* = 0.13). The most common solicited systemic AEs were headache (5) and arthralgia (5). No increase in AEs with successive doses was observed and only one subject reported a solicited systemic AE after the final dose. No subject in any group experienced an unsolicited AE that was considered related to immunization. There were no differences between any of the four groups with respect to the number or severity of AEs associated with vaccination.

**Table 2:** Solicited adverse events postvaccination (number of subjects experiencing AE)

	PfSPZ vaccine					Normal saline
	Group 1 (*N* = 21)	Group 2 (*N* = 21)	Group 3 (*N* = 21)	Group 4 (*N* = 21)	Total AE (*N* = 84)	Groups 1–4 (*N* = 20)
Any local solicited adverse event*	1 (4.8%)	3 (14%)	6 (29%)	2 (9.5%)	12 (14%)	2 (10%)
Tenderness	1 (4.8%)	3 (14%)	6 (29%)	1 (4.8%)	11 (13%)	1 (5%)†
Bruising	0	0	1 (4.8%)	0	1 (1.2%)	0
Swelling	0	0	0	1 (4.8%)	1 (1.2%)	0
Pruritus	0	0	0	0	0	1 (5%)‡
Any solicited systemic adverse event*	2 (9.5%)	3 (14%)	3 (14%)	1 (4.8%)	9 (11%)§	0§
Headache	0	1 (4.8%)	3 (14%)	1 (4.8%)	5 (6.0%)	0
Fatigue	0	1 (4.8%)	0	0	1 (1.2%)	0
Myalgia	1 (4.8%)	0	0	0	1 (1.2%)	0
Arthralgia	0	1 (4.8%)	2 (9.5%)	0	3 (3.6%)	0
Chills	0	0	1 (4.8%)	0	1 (1.2%)	0
Generalized pruritis	1 (4.8%)	0	0	0	1 (1.2%)	0

AE = adverse event; PfSPZ = *Plasmodium falciparum* sporozoites.

* Subjects may have more than one of the listed individual adverse events.

† Group 4.

‡ Group 1.

§ Comparison between vaccine and placebo for total AEs: *P* = 0.13, Barnard’s test, two-tailed.

#### Laboratory abnormalities following immunization.

There were no significant differences (Barnard’s test, two-tailed) in the number of subjects experiencing any laboratory abnormalities grade 2 or higher between vaccinees and controls. Grade 2 or higher laboratory abnormalities were reported in 18 vaccinees (21%) and seven controls (35%) (*P* = 0.22). The most commonly reported laboratory abnormality was increased eosinophils (15 vaccinees, six controls, *P* = 0.25), followed by increased AST (two vaccinees, one control, *P* = 0.69) and neutropenia (two vaccinees, zero controls, *P* = 0.60).

#### AEs after CHMI.

For solicited AEs reported on days 1–5 after CHMI, three subjects reported tenderness at the site of injection, one subject reported mild headache, and one reported diarrhea. For solicited AEs reported on days 6–28 after CHMI, those rated as grade 2 or 3 (11 AEs in six subjects) were all considered related to malaria as they correlated with the presence of parasitemia by TBS (all 11 AEs) and were highly correlated by time of initial presentation with the prepatent period by TBS (*r* = 0.87, *P* = 0.0019, Spearman correlation, two-tailed). Two subjects had grade 3 fever considered related to Pf infection—one vaccinee (fever on day 13, qPCR positive day 12.5, and TBS positive day 19) and one control (fever on day 18, qPCR positive day 13, and TBS positive day 19). In contrast, a grade 1 solicited AE on days 6–28 after CHMI corresponded to parasitemia by TBS in only 37 of 78 episodes (47%) and to parasitemia by qPCR in only 45 of 78 episodes, 58%).

## DISCUSSION

The EGSPZV3 study was designed to assess and compare vaccination regimens with a goal to identify the regimen with an acceptable safety profile and the highest vaccine-induced protection for future clinical development. We first selected the best regimen from the Warfighter 2 trial in the United States, ^15^ which consisted of four priming doses on days 1, 3, 5, and 7 and a delayed final dose at 16 weeks (Group 1 in the EGSPZV3 trial, Figure 1). This multi-dose prime regimen had provided approximately twice the protection compared with three other regimens studied in the same study, each of which consisted of three widely-spaced single injections also administered over 16 weeks. Vaccine efficacy was 40% against a stringent heterologous CHMI at 12 weeks (*P* = 0.04 using Barnard’s test) and higher than the 20%, 21%, and 23% VE at 12 or 24 weeks shown by the other regimens, none of which achieved statistical significance. ^15^ However, the regimen required five doses and took 16 weeks to complete, a complex approach that would be sub-optimal to implement in the field. A second regimen demonstrating the best protection in a regimen-comparison trial used a two-dose prime (days 1 and 8) and a 4-week final dose, an approach offering distinct advantages in speed and simplicity (Group 4 in the EGSPZV3 trial, Figure 1). The regimen provided 83% VE against heterologous CHMI at 9.5 weeks in the MAVACHE trial in Germany (NCT02704533) (Mordmüller, unpublished).

To best compare these two leading regimens, we included two additional groups: the four-dose prime from Warfighter 2 without a final dose (Group 2), to determine whether a final dose was needed, and the four-dose prime from Warfighter 2 with a 4-week rather than 16-week final dose (Group 3) to match the 4-week final dose in Group 4, based on the premise that a final booster dose might be needed but did not depend on a 16-week delay. We further decided to conduct the study in a malaria-exposed population, as this is the most important target population for field application of PfSPZ-based vaccines.

In the current study, of the four regimens evaluated, only the 1-, 8-, and 29-day regimen gave statistically significant VE (51.3%) against homologous CHMI at 6–7 weeks. Based on these results, this dose regimen has been down-selected for future Phases 2 and 3 testing in malaria naïve and malaria preexposed populations. An appealing aspect of this regimen is that it is completed in 4 weeks’ time, compared with earlier regimens that extended over 16–20 weeks. ^2^^,^ ^3^^,^ ^6^^–^ ^8^^,^ ^10^^,^ ^11^^,^ ^14^^,^ ^15^^,^ ^17^ This will increase the feasibility of MVPs where, to halt malaria transmission, entire populations need to be immunized as quickly and efficiently as possible.

It was unclear in this study why Group 4 outperformed Group 3, as the only difference between the two groups was a four-dose (Group 3) rather than a two-dose (Group 4) multi-dose prime. Recent data suggest that innate immune activation and specific myeloid signatures prevaccination diminish protection afforded by PfSPZ Vaccine in malaria-exposed African infants (Senkpeil, unpublished), and examination of whether four sequential doses might enhance these features is a subject for future study.

Although the EGSPZV3 trial met its overall objective, the analysis of VE in the four groups lost expected power because six of the 19 controls did not develop detectable Pf parasitemia after CHMI. In previous trials with PfSPZ Challenge (NF54) administered by DVI at a dose of 3,200 PfSPZ, parasitemia developed in 79/79 (100%) of malaria-naive control subjects ^20^^–^ ^29^ and in 35/35 limited malaria preexposed Tanzanian control subjects undergoing first CHMI ^8^^,^ ^10^ (Jongo, unpublished). Data from more heavily malaria-exposed populations provide a somewhat different picture, however. In our previous trial in Equatorial Guinea, ^14^ where malaria exposure is heavier than in Bagamoyo, Tanzania, 6/7 (86%) controls developed parasitemia by qPCR and 4/6 (67%) by TBS after CHMI. In a study in neighboring Gabon, among adults with normal hemoglobin (lacking sickle cell trait), 9/11 (82%) developed parasitemia by qPCR and 7/11 (64%) by TBS, and those with sickle cell trait were similar, with 7/9 (78%) developing parasitemia by qPCR, and 5/9 (56%) by TBS. ^24^ In a CHMI trial in Mali, where lifelong malaria exposure is particularly intense, using the same PfNF54 strain and 3,200 PfSPZ dose for CHMI as in the above studies, 8/15 (53%) control subjects developed Pf parasitemia by qPCR and only 1/15 (7%) had a positive TBS. ^17^ Thus, the control infection rate of 13/19 (68%) in our current study in Equatorial Guinea is not surprising.

We assessed antibodies to PfEXP1 and PfMSP1 prior to CHMI in order to determine why parasitemia did not develop in some controls. The uninfected control subjects had 6.4-fold higher antibody levels to PfEXP1 (*P* = 0.013) and 5.4-fold higher levels of antibodies to PfMSP1 (*P* = 0.046) than did the infected controls (Figure 5). This indicated that these uninfected subjects had naturally acquired immunity to Pf blood stage parasites that prevented them from developing parasitemia after administration of PfSPZ Challenge; three of the six controls who did not develop parasitemia had parasitemia detected by qPCR during the period of immunization, suggesting that infection acquired post artemether-lumefantrine pretreatment may have contributed to this naturally acquired immunity. The findings were similar in uninfected versus infected vaccinees following CHMI in the various vaccine groups, with uninfected vaccinees showing higher levels of antibodies to both PfEXP1 and PfMSP1 in all cases except for PfEXP1 in Group 3, and this difference achieved statistical significance for PfEXP1 in Group 2 (Figure 5). Another factor that might have affected results was sickle cell trait or other hemoglobinopathies, although these were not assessed based on the data provided above that the proportion developing parasitemia in Gabon following administration of PfSPZ Challenge (NF54) did not appear to be significantly influenced ^24^ by sickle cell trait. In Tanzania, alpha-thalassemia heterozygosity had no apparent effect on infectivity. ^35^

The VE seen against homologous CHMI in this trial was moderate. In the same study population in Equatorial Guinea, three doses of 2.7 × 10^6^ PfSPZ administered at 8-week intervals of PfSPZ Vaccine did not give significant VE (27%) against homologous CHMI at 15 weeks after last dose. ^14^ In the same earlier study, three doses of 1 × 10^5^ nonattenuated (fully infectious) PfSPZ administered at 4-week intervals under cover of chloroquine (PfSPZ-CVac approach) conferred significant 55% VE against homologous CHMI at 14 weeks after the last dose despite using 9-fold fewer PfSPZ (3 × 10^5^ versus 2.7 × 10^6^ in Group 4). These data are consistent with the greater potency of PfSPZ-CVac in malaria-naive populations. ^22^^,^ ^37^

In the current trial, there was no correlation between antibodies to PfCSP 2 weeks after the last dose of vaccine or just prior to CHMI and protection status (Figure 4). Antibody levels 2 weeks after the day 7 (fourth) dose were similar in Groups 1–3 (Supplemental Figure 1) and did not increase further post final dose in Groups 1 or 3. Furthermore, at the time of CHMI, Group 1 had significantly lower PfCSP antibody levels than Groups 2 and 3, a finding for which we have no explanation (Figure 4 and Supplemental Figure 1). Correlations between anti-PfCSP antibody levels (post-vaccination or pre-CHMI) and protection have been identified in some but not all prior CHMI studies in malaria-exposed African adults immunized with PfSPZ Vaccine, ^8^^,^ ^11^^,^ ^14^ and this inconsistency may be the product of the small sample sizes used in these studies. In contrast, when PfSPZ Vaccine has been evaluated for protection against naturally transmitted Pf malaria in the field, larger sample sizes have been studied, and have consistently shown statistically significant differences in the anti-PfCSP antibody responses (or the fold rise in antibody responses) between those infected and those uninfected during the follow-up period (Sirima and Laurens, unpublished ^7^^,^ ^17^). Because liver resident CD8 T cells are thought to underlie the protection induced by PfSPZ Vaccine but cannot be measured in the periphery, ^3^ the anti-PfCSP antibody results in field protection studies have been interpreted to be a marker for underlying cellular responses rather than a protective mechanism *per se*. It is possible that stronger associations between anti-PfCSP antibodies and protection would have been found in the current study had larger sample sizes been evaluated. At this point, it is not known how multi-dose priming may affect antibody or cellular responses to PfSPZ Vaccine.

PfSPZ Vaccine administered by DVI was safe and very well tolerated in all four regimens. Mild headache and mild arthralgia were the most commonly reported systemic AEs, but the frequency was not statistically different between vaccinees and controls or between study groups 1–4. There were no grade 3 AEs, no grade 3 laboratory abnormalities, and no SAEs attributed to administration of PfSPZ Vaccine.

Administration of PfSPZ Challenge for CHMI in this malaria-exposed adult population, using TBS positivity to initiate treatment of parasitemia resulting from CHMI, was also safe and well tolerated. There were very few AEs in the 5 days after inoculation, and these were all mild in severity. As in other studies in Africa and our previous experience in Equatorial Guinea, symptoms and signs consistent with malaria (those occurring on or after day 6, the time of first emergence of parasites from the liver into the bloodstream) were minimal. ^8^^,^ ^11^^,^ ^14^^,^ ^17^^,^ ^24^^,^ ^35^^,^ ^38^^–^ ^40^ Six of 47 subjects developing parasitemia experienced grade 2 AEs, two of whom concurrently developed grade 3 fevers. All grade 2 and 3 AEs were associated with Pf parasitemia detected by TBS and the onset of grade 2 and 3 AEs was highly correlated with the time of positive TBS. In contrast, grade 1 AEs appeared to reflect background rates in the community as they occurred with equal frequency in those who did not develop parasitemia.

In summary, we were encouraged that significant VE was demonstrated with an immunization regimen of 9.0 × 10^5^ PfSPZ on days 1, 8, and 29. Achieving optimal VE is challenged by the effects of current and previous infections with Pf and possibly other *Plasmodium* species circulating on Bioko Island. ^36^ To better understand and define VE and these confounders, further studies with this regimen are planned or underway in the United States, Equatorial Guinea, Germany, and Mali in populations with the degree of prior Pf exposure varying according to the area of residence and the participant’s age.

## Supplemental Material


Supplemental materials
