# Supplementary material for: Multi-Dose Priming Regimens of PfSPZ Vaccine: Safety and Efficacy against Controlled Human Malaria Infection in Equatoguinean Adults
Source: Am J Trop Med Hyg. 2022 Feb 7;106(4):1215–26. doi: 10.4269/ajtmh.21-0942 (PMC8991366; doi:10.4269/ajtmh.21-0942)
Supplement: Supplementary file 1 [file tpmd210942.SD1.pdf]

Supplemental Table 1: Demographics

		Group 1(9.0x10^5 - Day 1, 3, 5, 7, 113)		Group 2(9.0x10^5 - Day 1, 3, 5, 7)		Group 3(9.0x10^5 - Day 1, 3, 5, 7, 29)		Group 4(9.0x10^5 - Day 1, 8, 29)	
		**Vaccine (N=21)**	**Placebo (N=5)**	**Vaccine (N=21)**	**Placebo (N=5)**	**Vaccine (N=21)**	**Placebo (N=5)**	**Vaccine (N=21)**	**Placebo (N=5)**
Sex – n (%)	Male	21 (100)	5 (100)	17 (81)	5 (100)	18 (86)	5 (100)	15 (71%)	5 (100%)
	Female			4 (19)		3 (14)		6 (29%)	0
Age	Mean (SD)	23.3 (2.47)	25.2 (1.92)	21.9 (2.85)	22.0 (5.83)	23.9 (3.46)	20.6 (2.07)	25.0 (3.36)	24.2 (1.30)
	Median	23	25	21	20	24	21	26	24
	Minimum, Maximum	18,30	23,28	18,26	18,32	18,31	18,23	19,30	23,26
Height (cm)	Mean (SD)	168 (6.31)	164 (3.05)	167 (5.78)	169 (6.58)	167 (5.58)	172 (7.44)	168 (8.66)	172 (3.63)
	Median	168	163	167	168	167	171	166	172
	Minimum, Maximum	156,180	160,168	156,179	161,177	156,179	162,181	153,181	166,176
Weight (kg)	Mean (SD)	66.2 (10)	65.4 (4.5)	64.5 (8.0)	66.6 (10)	63.6 (8.3)	71.8 (7.8)	64.3 (9.0)	68.1 (7.0)
	Median	63.2	67.5	64.8	71	62	73	65	66
	Minimum, Maximum	48.2,91.7	60.2,70.5	49,79	56,79	51.5,86	60,80.6	50.7,82	59,76.2
BMI	Mean (SD)	23.2 (2.55)	24.4 (2.41)	23.0 (2.32)	23.2 (2.39)	22.9 (2.43)	24.0 (1.00)	22.7 (2.67)	23.2 (2.17)
	Median	23	25	23	23	23	24	23	24
	Minimum, Maximum	19,28	21,27	18,26	20,26	18,30	23,25	19,29	20,25

Supplemental Table 2: Asymptomatic parasitemia during the study prior to CHMI detected retrospectively by qPCR*.

Group	Vaccine or placebo	Vaccination Visit	Parasite density (copies/µL)	TBS results - CHMI	qPCR results - CHMI
1	PfSPZ	1 day prior to V1	1.31	negative	negative
3	NS	1 day prior to V5	16.31	negative	negative
3	NS	1 day prior to V1	1.84	negative	negative
3	NS	1 day prior to V5	227.4	negative	negative
4	NS	1 day prior to planned V3**	6740	negative	Positive day 20
4	PfSPZ	1 day prior to V3	0.39	negative	negative
4	PfSPZ	7 days prior to V3	0.70	negative	negative

*All subjects were treated as per protocol prior to V1 and prior to CHMI, none specifically for qPCR positivity.

** This subject was the only subject who was TBS + (709 Pf/µL). This subject did receive treatment and therefore V3 was delayed by 20 days after planned date, with CHMI 6 weeks later.

Notes: The five subjects positive at screening are not included in the table as all subjects received AL prior to immunization (see manuscript text); qPCR samples from all other timepoints including the pre-CHMI timepoint were negative for these seven subjects.

Supplemental Table 3. Antibodies to PfCSP, PfMSP1 and PfEXP1. Out-of-range values are reported as 1.

Group	Volunteer ID	Infection	PfCSP IgG ELISA										PfMSP1	PfEXP1
			V1-1	V4+14	Net	Ratio	V5+14	Net	Ratio	CHMI-7	Net	Ratio		
Group 1: 9.0x105 PfSPZ administered four times on Days 1, 3, 5 and 7 (0, +2, +4, +6 days) as a prime, followed by a boost of 9.0x105 PfSPZ on Day 113 (+16 weeks)	ANN-001	Uninfected	1538	3063	1525	1.99	21804	20266	14.18	22183	20645	14.42	267	324
	GAO-007	Uninfected	1109	17114	16005	15.43	5757	4648	5.19	8136	7027	7.34	3636	311
	FMS-011	Uninfected	223	1570	1347	7.04	772	549	3.46	543	320	2.43	450	383
	DMM-013	Uninfected	566	74251	73685	131.19	31773	31207	56.14	29029	28463	51.29	2648	1234
	SNB-017	Uninfected	1219	6388	5169	5.24	3030	1811	2.49	4058	2839	3.33	2336	3234
	JMA-015	Uninfected	56	13115	13059	234.20	2887	2831	51.55	1782	1726	31.82	64	189
	FEN-028	Uninfected	852	3422	2570	4.02	2097	1245	2.46	1667	815	1.96	1003	1037
	JOA-019	Uninfected	344	1273	929	3.70	557	213	1.62	619	275	1.80	498	567
	MMM-032	Uninfected	138	1635	1497	11.85	1625	1487	11.78	1168	1030	8.46	1942	1684
	004	Uninfected	177	40506	40329	228.85	3588	3411	20.27	4532	4355	25.60	114	386
	NMA-027	Infected	382	7529	7147	19.71	2279	1897	5.97	1340	958	3.51	1418	848
	LEE-024	Infected	212	40317	40105	190.17	9703	9491	45.77	8480	8268	40.00	287	398
	JOM-025	Infected	91	8050	7959	88.46	2856	2765	31.38	1674	1583	18.40	961	209
	ROB-018	Infected	25	22537	22512	901.48	8994	8969	359.76	7390	7365	295.60	281	2204
	MSE-021	Infected	410	22233	21823	54.23	3240	2830	7.90	2965	2555	7.23	21	71
	SNB-023	Infected	113	63810	63697	564.69	15645	15532	138.45	7800	7687	69.03	1	19
	ESB-034	Infected	534	14499	13965	27.15	6329	5795	11.85	3512	2978	6.58	2233	219
Group 1: Normal Saline Placebo	NEN-31	Uninfected	NS	556	555	555.00	737	736	737.00	346	345	346.00	3625	8545
	EEN-014	Uninfected	738	626	1	0.85	362	1	1.00	428	1	1.00	1817	5641
	RBM-030	Infected	774	NS	NS	NS	285	1	1.00	439	1	1.00	1716	1665
	COO-002	Infected	667	383	1	0.57	212	1	1.00	207	1	1.00	240	213
	JBC-010	Infected	80	61	1	0.76	NS	NS	NS	95	15	1.19	12050	310
Group 2: 9.0x105 PfSPZ administered four times on Days 1, 3, 5 and 7 (0, +2, +4, +6 days) as a prime, without a boost	MNC-040	Uninfected	516	131469	130953	254.78	N/A			29458	28942	57.09	2424	2350
	JOE-051	Uninfected	217	5493	5276	25.31				4692	4475	21.62	1032	729
	PPT-047	Uninfected	14	49434	49420	3531.00				6898	6884	492.71	205	18
	MBA-062	Uninfected	982	7146	6164	7.28				24851	23869	25.31	878	794
	OCB-065	Uninfected	20	9360	9340	468.00				4214	4194	210.70	1	1
	VAO-072	Uninfected	580	15201	14621	26.21				7515	6935	12.96	320	30751
	CBN-082	Uninfected	91	7170	7079	78.79				12728	12637	139.87	59	2896
	MNN-060	Uninfected	1686	9050	7364	5.37				24962	23276	14.81	2090	524
	ROA-099	Uninfected	734	18886	18152	25.73				2348	1614	3.20	38848	4348
	AMM-097	Uninfected	1271	33995	32724	26.75				10347	9076	8.14	1595	1845
	BST-102	Uninfected	235	803	568	3.42				1066	831	4.54	6252	2394
	ANA-043	Infected	10	3440	3430	344.00				6747	6737	674.70	326	55
	REC-057	Infected	1126	13414	12288	11.91				9003	7877	8.00	153	1
	JSB-038	Infected	1	18857	18856	18857.00				2461	2460	2461.00	1	103

Group	Volunteer ID	Infection	PfCSP IgG ELISA										PfMSP1	PfEXP1
			V1-1	V4+14	Net	Ratio	V5+14	Net	Ratio	CHMI-7	Net	Ratio		
	EEN-039	Infected	673	57640	56967	85.65				32181	31508	47.82	70	59
	BRB-073	Infected	77	16279	16202	211.42				9442	9365	122.62	1	81
	JPL-066	Infected	1	73379	73378	73379.00				4085	4084	4085.00	1	134
	EBB-067	Infected	187	3844	3657	20.56				2169	1982	11.60	227	592
	RFM-035	Infected	1	55663	55662	55663.00				5561	5560	5561.00	7831	250
	RNO-090	Infected	3687	9595	5908	2.60				19765	16078	5.36	1941	1600
	DLB-053	Infected	1	46399	46398	46399.00				5890	5889	5890.00	40	67
**Group 2: Normal Saline Placebo**	MBJ-050	Infected	185	187	2	1.01				32	1	1.00	133	141
	PNN-084	Infected	1437	323	1	1.00				427	1	1.00	822	1360
	OEI-077	Infected	49	104	55	2.12				262	213	5.35	447	195
	SOO-096	Infected	1	129	128	129.00				32	31	32.00	43	149
**Group 3: 9.0x105 PfSPZ administered four times on Days 1, 3, 5 and 7 (0, +2, +4, +6 days) as a prime, followed by a boost of 9.0x105 PfSPZ on Day 29 (+4 weeks)**	MOB-058	Uninfected	130	*158933*	158803	1222.56	43096	42966	331.51	24910	24780	191.62	886	329
	ENM-125	Uninfected	892	6210	5318	6.96	6131	5239	6.87	5203	4311	5.83	5747	1050
	CMM-116	Uninfected	186	8160	7974	43.87	3581	3395	19.25	2582	2396	13.88	1225	811
	DNO-126	Uninfected	431	5546	5115	12.87	3667	3236	8.51	4296	3865	9.97	1299	611
	MAE-137	Uninfected	172	30353	30181	176.47	16639	16467	96.74	14219	14047	82.67	154	25
	PNA-118	Uninfected	15996	33779	17783	2.11	23307	7311	1.46	13044	-2952	0.82	167267	41545
	FNN-136	Uninfected	637	47537	46900	74.63	26003	25366	40.82	27290	26653	42.84	590	5945
	AMN-120	Uninfected	1512	35161	33649	23.25	26711	25199	17.67	22628	21116	14.97	1765	11722
	AEB-141	Uninfected	491	6657	6166	13.56	6648	6157	13.54	6133	5642	12.49	332	153
	PNA-119	Infected	3843	16909	13066	4.40	9806	5963	2.55	5770	1927	1.50	12168	4556
	VBM-129	Infected	2201	13755	11554	6.25	10636	8435	4.83	6977	4776	3.17	102	904
	JNN-117	Infected	704	7629	6925	10.84	4723	4019	6.71	3238	2534	4.60	722	1074
	ALB-130	Infected	109	15098	14989	138.51	15374	15265	141.05	10945	10836	100.41	127	1777
	TNN-135	Infected	680	19341	18661	28.44	13510	12830	19.87	8988	8308	13.22	1127	11147
	POB-063	Infected	109	57184	57075	524.62	30997	30888	284.38	17364	17255	159.30	78	1
	OMA-114	Infected	323	11736	11413	36.33	7342	7019	22.73	4749	4426	14.70	52	267
	MBM-055	Infected	71	12158	12087	171.24	6575	6504	92.61	5964	5893	84.00	419	54
	JSB-109	Infected	510	6524	6014	12.79	8458	7948	16.58	10849	10339	21.27	2895	193
	CBE-122	Infected	249	50288	50039	201.96	26193	25944	105.19	23328	23079	93.69	692	1204
	JPL-138	Infected	286	29755	29469	104.04	18887	18601	66.04	11222	10936	39.24	7	669
**Group 3: Normal Saline Placebo**	JAE-134	Infected	401	406	5	1.01	874	473	2.18	839	438	2.09	14645	3966
	FEN-104	Infected	509	380	1	1.00	375	1	1.00	345	1	1.00	1017	1954
	ANN-016	Infected	156	136	1	1.00	88	1	1.00	138	1	1.00	2155	1223
	ANB-128	Uninfected	521	320	1	1.00	324	1	1.00	210	1	1.00	349	87
	ABB-106	Uninfected	83	128	45	1.54	46	1	1.00	124	41	1.49	212	456
**Group 4: 9.0x105 PfSPZ administered two times on Days 1 and 8 (0, +7 days) as a**	MAM-069	Uninfected	222	4184	3962	18.85	N/A			528	306	2.38	1950	736
	RMA-146	Uninfected	179	14748	14569	82.39				3642	3463	20.35	520	468
	ANA-166	Uninfected	269	4807	4538	17.87				5268	4999	19.58	5424	2641
	VAB-150	Uninfected	354	7795	7441	22.02				7945	7591	22.44	838	1502

Group	Volunteer ID	Infection	PfCSP IgG ELISA										PfMSP1	PfEXP1
			V1-1	V4+14	Net	Ratio	V5+14	Net	Ratio	CHMI-7	Net	Ratio		
prime, followed by a boost of 9.0x105 PfSPZ on Day 29 (+4 weeks)	EOM-078	Uninfected	829	3347	2518	4.04				4551	3722	5.49	1077	886
	REA-173	Uninfected	1492	13932	12440	9.34				28259	26767	18.94	468	15981
	AMM-157	Uninfected	1179	2830	1651	2.40				4300	3121	3.65	238	2930
	MEM-076	Uninfected	63	47133	47070	748.14				32717	32654	519.32	1	68
	SAO-154	Uninfected	288	4336	4048	15.06				4013	3725	13.93	9537	563
	MNC-070	Uninfected	255	32711	32456	128.28				18864	18609	73.98	1	159
	DLM-071	Uninfected	203	17784	17581	87.61				9184	8981	45.24	167	160
	JON-151	Uninfected	337	965	628	2.86				2879	2542	8.54	13213	8321
	AOM-107	Uninfected	530	6372	5842	12.02				107697	107167	203.20	58001	24381
	BNN-101	Uninfected	337	42854	42517	127.16				2546	2209	7.55	15791	4468
	RON-158	Infected	209	21467	21258	102.71				13547	13338	64.82	48	433
	RSS-091	Infected	22	20803	20781	945.59				16547	16525	752.14	1	19
	EMS-171	Infected	137	14985	14848	109.38				16021	15884	116.94	1394	2580
	JAO-148	Infected	447	4189	3742	9.37				1079	632	2.41	2124	1389
	PNM-161	Infected	179	3996	3817	22.32				10685	10506	59.69	1126	963
	DRB-080	Infected	1	5560	5559	5560.00				17151	17150	17151.00	947	350
	VAM-168	Infected	50	2699	2649	53.98				864	814	17.28	88	72
Group 4: Normal Saline Placebo	AAM-162	Uninfected	207	965	758	4.66				229	22	1.11	938	414
	SEA-163	Infected	89	206	117	2.31				87	1	1.00	263	1715
	PMA-149	Infected	960	104	1	1.00				454	1	1.00	2976	644
	AON-139	Infected	1651	524	1	1.00				2135	484	1.29	153	1242
	BEO-155	Infected	412	1287	875	3.12				661	249	1.60	6288	905

NS: No samples available

Supplemental Table 4: Inclusion Criteria

1. Healthy males and non-pregnant/non-lactating females, age 18 to 45 years at time of enrollment.
2. Provision of signed and dated informed consent form.
3. Demonstrate understanding of the study by responding correctly to 10 out of 10 true/false statements about the trial (a maximum of two additional attempts will be granted for those who fail to respond correctly to all true/false statements in their first attempt).
4. Stated availability and willingness to comply with all study procedures and visits for the duration of the trial, including the required vaccination and post-CHMI ward observation period.
5. Able to understand and communicate in Spanish, the national language of Equatorial Guinea
6. Be in good general health as evidenced by medical history, screening physical examination and laboratory findings.
7. Females of child bearing potential must agree to use injectable medroxyprogesterone for at least 4 weeks prior to enrollment and agree to continue to use medroxyprogesterone during the entire study period.
8. Female subjects must not be pregnant (as demonstrated by a negative urine pregnancy test) at enrollment and prior to each immunization.
9. Body Mass Index (BMI) of 18 to 30 kg/m^2.
10. At least one year of residence on Bioko Island, Equatorial Guinea, and living close enough to Baney Clinical Research Center and Sampaka Hospital to be able to attend the required appointments at the study center.
11. Agree to release medical information and inform a study doctor about contraindications for participation in the study.
12. Willingness to be attended to by a study doctor and take all medications prescribed during the study period.
13. Agree to provide contact information of a third-party household member or close friend to the study team.
14. Agree not to participate in another clinical trial during the study period.
15. Agree not to donate blood during the study period.
16. Willing to undergo HIV, hepatitis B (HBV) and hepatitis C (HCV) tests.
17. Reachable by telephone for adverse event review.

Supplemental Table 5: Exclusion Criteria

1. Known allergic reactions to components of PfSPZ Vaccine, PfSPZ Challenge, or artemether/lumefantrine (AL).
2. Having received an investigational malaria vaccine in the last 5 years.
3. Having received any non-live vaccine in the 14 days prior to enrollment, any live vaccine in the 28 days prior to enrollment or three or more of any type of vaccine in the four months prior to enrollment.
4. Participation in any other clinical study involving investigational medicinal products including malaria drugs within 30 days prior to enrollment.
5. History of arrhythmias, prolonged QT-interval or other cardiac disease, or clinically significant abnormalities on electrocardiogram (ECG) at screening.
6. History of non-febrile seizures or complex febrile seizures.
7. History of cardiac disease in a 1st or 2nd degree relative when <50 years of age.
8. A chronic illness including diabetes mellitus, cancer, HIV/AIDS, tuberculosis.
9. History of illicit drug or alcohol use that interferes with normal social function.
10. The use of chronic immunosuppressive drugs or other immune modifying drugs within three months of study onset (inhaled and topical corticosteroids are allowed) and during the study period.
11. Any clinically significant deviation from the normal range in biochemistry, hematology or urinalysis tests.
12. Positive HIV, hepatitis B virus or hepatitis C virus serologic tests.
13. Signs and symptoms of tuberculosis (e.g., chronic cough, night sweats, chronic fever, enlarged lymph nodes, unintended weight loss), or risk factors in an otherwise healthy person in combination with a positive tuberculin skin test (TST).
14. Symptoms, physical signs and/or laboratory values suggestive of systemic disorders including renal, hepatic, blood, cardiovascular, pulmonary, skin, immunodeficiency, psychiatric, and other conditions which could interfere with the interpretation of the study results or compromise the health of the volunteer.
15. Any medical, psychiatric, social or occupational condition or situation that, in the judgment of the PI, impairs the volunteer’s ability to give informed consent, increases the risk to the volunteer of participation in the study, affects the ability of the volunteer to participate fully in the study, or might negatively impact the quality, consistency or interpretation of data derived from their participation in the study.

Supplemental Table 6: List of solicited adverse events with the grading system for severity and grading for relatedness.

Local solicited adverse events<br><br>(at injection site)	• Pain<br>• Tenderness<br>• Pruritus	1	Daily activity minimally affected, with or without treatment
		2	Daily activity possible but only with treatment
		3	Daily activity not possible even with treatment
	• Erythema<br>• Swelling<br>• Induration<br>• Bruising/extravasated blood	1	2.5 – 5 cm
		2	5.1 – 10 cm
		3	>10 cm, necrosis or exfoliative dermatitis
Systemic solicited adverse events<br><br>(Core List-Post Vaccination)	• Fever	1	38.0°C – 38.4°C
		2	38.5°C – 38.9°C
		3	>39.0°C
	• Allergic reaction (rash, urticaria, pruritus, edema)<br>• Headache<br>• Subjective Fever*<br>• Fatigue<br>• Malaise<br>• Chills<br>• Myalgia<br>• Arthralgia	1	Daily activity minimally affected, with or without treatment
		2	Daily activity possible but only with treatment
		3	Daily activity not possible even with treatment
Post CHMI malaria symptoms<br><br>(In addition to Core List)	• Dizziness<br>• Rigors (body shaking shivers)<br>• Sweats<br>• Cough<br>• Nausea<br>• Vomiting	1	Daily activity minimally affected, with or without treatment
		2	Daily activity possible but only with treatment
		3	Daily activity not possible even with

	• Abdominal pain<br>• Diarrhea<br>• Chest pain<br>• Palpitations<br>• Shortness of breath		treatment

Definitely Related – The investigational product is the only plausible etiology.
Probably Related – The investigational product is the most plausible etiology, but there is at least one other etiology that is reasonably likely.
Possibly Related – The investigational product is not the most plausible etiology, but is still reasonably likely.
Unlikely to be related – The investigational product is unlikely to be the cause, but cannot be completely ruled out due to the existence of plausible theoretical mechanisms linking the investigational product to the adverse event.
Not Related – There is no plausible theoretical mechanism whereby the investigational product could have caused the adverse event

Supplemental Table 7: Severity grading scale for biochemistry and hematology.

TOXICITY GRADING SCALE FOR GRADING CLINICAL LABORATORY PARAMETERS; HEALTHY ADULT AND ADOLESCENT VOLUNTEERS (11-65 YEARS OLD)							
**HEMATOLOGY PARAMETERS**	Source	Units	Normal range	Mild (Grade1)	Moderate (Grade 2)	Severe (Grade 3)	Potentially life Threatening Grade (4)
Hemoglobin (Male)[1]	Local	g/dL	12 - 17.4	<12-8.0	<8.0 – 7.0	<7.0 – 4.0	<4.0
Hemoglobin (Female)[2]	Local	g/dL	9.6 - 14.1	<9.6 – 8.0			
Leukocyte count (WBC) Increase[1]	Local	x10^3/µL	3.65 - 9.7	>9.7 - 15	>15 - 20	>20 - 25	> 25
Leukocyte count (WBC) Decrease[1]	Local	x10^3/µL	3.65 - 9.7	<3.65 - 2.5	<2.5 - 1.5	<1.5 - 1	< 1
Lymphocytes Decrease[2]	Local	x10^3/µL	1.19 - 3.4	<1.19 - 0.75	<0.75 - 0.5	< 0.5 - 0.25	< 0.25
Neutrophils Decrease[1]	Local	x10^3/µL	1.61-5.69	<1.61 - 0.9	< 0.9 - 0.6	< 0.6 - 0.4	<0.4
Eosinophils - cell/mm3[(2)]	Local	x10^3/µL	0 - 0.78	> 0.78 - 1.5	>1.5 - 5	> 5	Hypereosinophilic
Platelets Decreased[1]	Local	x10^3/µL	124-312	75 - <124	50 - <75	25 – <50	< 25

TOXICITY GRADING SCALE FOR GRADING CLINICAL LABORATORY PARAMETERS; HEALTHY ADULT AND ADOLESCENT VOLUNTEERS (11-65 YEARS OLD)							
**BIOCHEMISTRY PARAMETERS**	Data Source	Units	Normal range	Mild (Grade1)	Moderate (Grade 2)	Severe (Grade 3)	Potentially life Threatening Grade (4)
Glucose – Hypoglycemia[2]	LOCAL	mg/dL	70-109	64- <70	56- <64	45- <56	<45
Random Glucose – Hyperglycemia[2]	LOCAL	mg/dL	70-109	>109-126.0	>126-198	> 198	Insulin requirement or hyperosmolar coma
Creatinine[6]	LOCAL	mg/dL	0.54-1.11	>1.11-1.70	>1.70-2.0	>2.0-2.5	> 2.5 or require dialysis
Albumin – Hypoalbuminemia[2]	LOCAL	g/L	42.7 - 60	28 - < 42.7	25 - < 28	< 25	∞
Total Protein – Hypoproteinemia Saathof, et al.[5]	LOCAL	g/L	67.2 - 85.2	55 - <67.2	50 - < 55	< 50	∞
Alkaline Phosphatase[5]	LOCAL	U/L	45- 186.9	> 186.9 – 356.9	>356.9 – 526.9	>526.9- 1700	>1700
Alanine aminotransferase[6]	LOCAL	U/L	0 - 45	>45 – 106.5	>106.5 – 209	>209 - 410	>410
Aspartate aminotransferase[5]	LOCAL	U/L	15.2 – 58.7	>58.7-138.8	>138.8-272.3	>272.3-534	>534
Bilirubin – when	LOCAL	mg/dL	0.18-1.82	>1.82-2.27	>2.27-2.73	>2.73-3.18	>3.18

	accompanied by any increase in Liver Function Test[2]							
	Bilirubin – when Liver Function Test is normal[5]	LOCAL	mg/dL	0-2.62	>2.62-3.01	>3.01-3.61	>3.612-4.19	>4.19

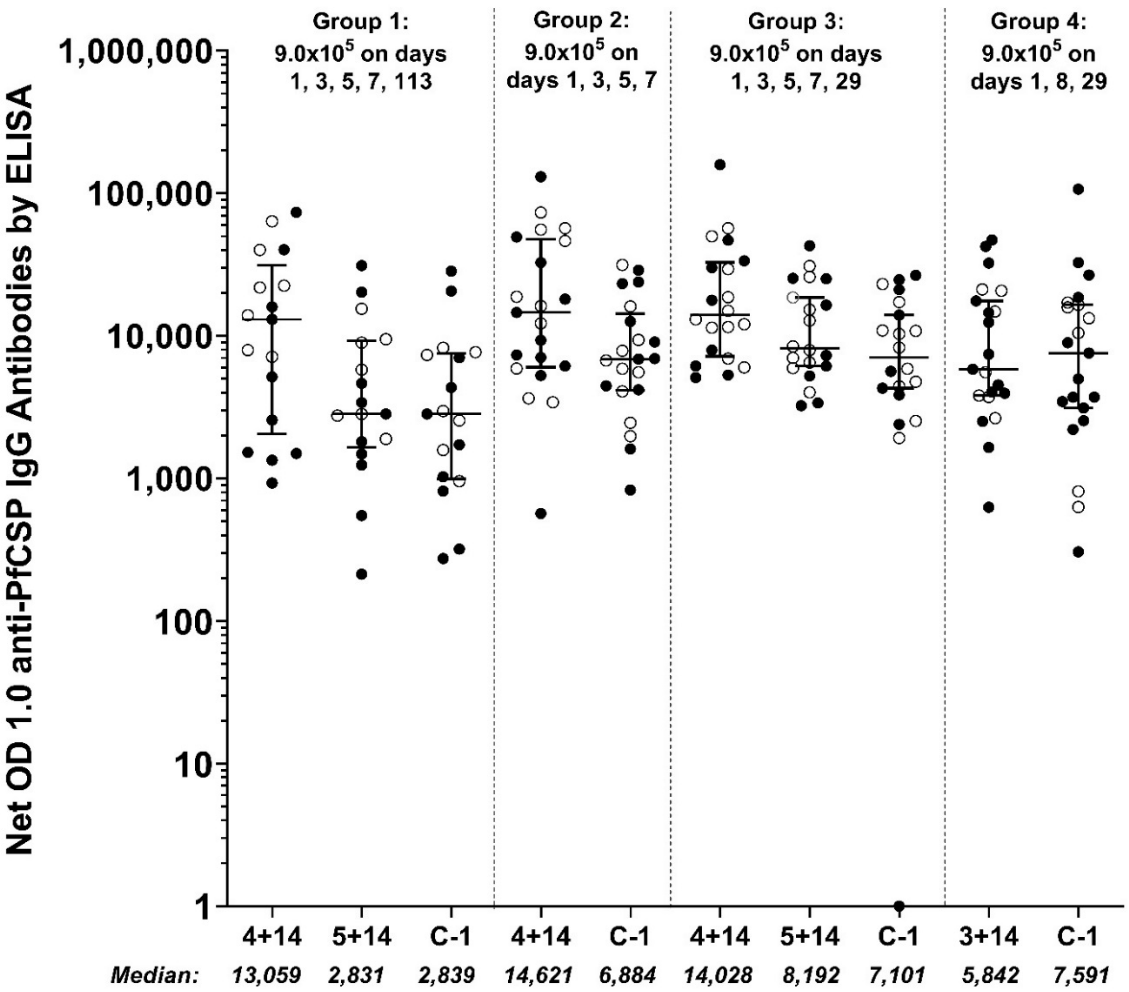

Supplemental Figure 1. PfCSP IgG Kinetics. Each point represents a subject infected (○) or not infected (●) after CHMI. For groups 1, 2 and 3 the highest antibody levels were achieved after the 4th dose (4+14), with no increase in antibody levels in groups 1 and 3 with a 5th dose (5+14). For subjects receiving a delaysed final dose (groups 1, 3 and 4) there was little decline in antibody level prior to CHMI 4-5 weeks later (C-1).
